# The Neural Basis of Individual Differences in Directional Sense

**DOI:** 10.3389/fnhum.2018.00410

**Published:** 2018-10-25

**Authors:** Heather Burte, Benjamin O. Turner, Michael B. Miller, Mary Hegarty

**Affiliations:** ^1^Department of Psychology, Tufts University, Medford, MA, United States; ^2^Wee Kim Wee School of Communication and Information, Nanyang Technological University, Singapore, Singapore; ^3^Department of Psychological & Brain Sciences, University of California, Santa Barbara, Santa Barbara, CA, United States

**Keywords:** spatial cognition, navigation, allocentric headings, sense-of-direction, individual differences

## Abstract

Individuals differ greatly in their ability to learn and navigate through environments. One potential source of this variation is “directional sense” or the ability to identify, maintain, and compare allocentric headings. Allocentric headings are facing directions that are fixed to the external environment, such as cardinal directions. Measures of the ability to identify and compare allocentric headings, using photographs of familiar environments, have shown significant individual and strategy differences; however, the neural basis of these differences is unclear. Forty-five college students, who were highly familiar with a campus environment and ranged in self-reported sense-of-direction, underwent fMRI scans while they completed the Relative Heading task, in which they had to indicate the direction of a series of photographs of recognizable campus buildings (i.e., “target headings”) with respect to initial “orienting headings.” Large individual differences were found in accuracy and correct decision latencies, with gender, self-reported sense-of-direction, and familiarity with campus buildings all predicting task performance. Using linear mixed models, the directional relationships between headings and the experiment location also impacted performance. Structural scans revealed that lateral orbitofrontal and superior parietal volume were related to task accuracy and decision latency, respectively. Bilateral hippocampus and right presubiculum volume were related to self-reported sense-of-direction. Meanwhile, functional results revealed clusters within the superior parietal lobule, supramarginal gyrus, superior frontal gyrus, lateral orbitofrontal cortex, and caudate among others in which the intensity of activation matched the linear magnitude of the difference between the orienting and target headings. While the retrosplenial cortex and hippocampus have previously been implicated in the coding of allocentric headings, this work revealed that comparing those headings additionally involved frontal and parietal regions. These results provide insights into the neural bases of the variation within human orientation abilities, and ultimately, human navigation.

## Introduction

Remaining oriented within environmental-scale spaces—environments that are too large to be viewed from one vantage point (Montello, 1993)—is essential for navigating through a city, pointing to unseen landmarks, and giving directions. While “being oriented” tends to be associated with knowing your current physical facing direction in relation to the environment (i.e., allocentric heading), we propose that knowing how imagined facing directions are related to the environment and how those imagined facing directions relate to other imagined facing directions or your current facing direction is also important for navigation tasks such as planning a route or giving directions. For instance, when giving directions, you need to know the facing direction of your addressee and imagine how that facing direction changes while traveling to ensure that their final facing direction leads them to their destination. Without being able to recall and compare imagined orientations with respect to environmental reference frames, it is impossible to provide accurate directions. We refer to this broader phenomenon of knowing your facing direction, imagining facing directions, and comparing facing directions as “directional sense.” Directional sense is not a sense like vision or audition, but depends on several cues, which include visual cues and self-motion perception (Wolbers and Hegarty, 2010).

It is well known that individuals vary in their environmental-scale spatial abilities (e.g., Hegarty et al., 2006; Ishikawa and Montello, 2006; Weisberg et al., 2014), but the underlying cause of this variation is not well understood. We propose that variation in directional sense may be a major factor in variation in environmental-scale spatial abilities, because environmental-scale tasks implicitly require directional sense. For instance, pointing toward an unseen landmark requires coordinating one’s facing direction with the direction to the location of the landmark. Accurate pointing cannot occur without knowing your physical (or imagined) facing direction with respect to the larger environment.

Not only are individuals variable in their environmental-scale spatial abilities, but they are also aware of their relative capacity in this regard, and are quite accurate in their self-reports of their abilities. Self-reported sense-of-direction is related to pointing toward unseen locations, distance estimation (Kozlowski and Bryant, 1977), pointing in a familiar environment, spatial updating, and learning spatial layouts (Hegarty et al., 2002). We propose that self-reported sense-of-direction maybe predictive of directional sense, due to its relationship to environmental-scale spatial abilities.

In the rest of the introduction, we describe previous work that has examined aspects of how the abilities underlying navigation are represented in the brain, alongside behavioral findings from previous studies using tasks related to the one we employ here. Although there is a substantial amount of evidence regarding different components of environmental-scale spatial abilities, no prior work has investigated individual differences in this ability at the neural level. In this paper, we investigate (1) the factors that contribute to variation in directional sense, including self-reported sense-of-direction, (2) variation in brain structure related to variation in directional sense, and (3) the neural basis of directional sense.

### Animal and Human Models of Allocentric Headings

Head-direction cells, which are the neurological basis of an organism’s ability to determine its facing direction (Taube, 1998), were first identified in rodents (Ranck, 1984). Originally, head-direction cells were identified in the dorsal region of a rodent’s presubiculum (Ranck, 1984), but they have subsequently been identified in a set of interconnected regions (see Sharp et al., 2001 for a review). Each head-direction cell fires whenever the animal faces the cell’s preferred direction (Taube et al., 1990a), which is grounded in the environment, that is, an allocentric-heading (Taube, 1998; as opposed an egocentric bearing, which is a direction relative to the axis of orientation of an organism, see Klatzky, 1998). Each cell shows a directional tuning function centered at the cell’s preferred direction, such that the cell’s maximal firing rate forms the peak of a Gaussian function (Taube et al., 1990a). As a collective group, head-direction cells form an attractor network of excitatory connections with cells that prefer nearby directions and inhibitory connections with cells that prefer distant directions (Sharp et al., 2001). The attractor network ensures that the head-direction system cannot code two facing directions simultaneously (Sharp et al., 2001). Familiar visual cues can reset the directional coding (Taube et al., 1990b).

In humans, the hippocampus has been conceptualized as the site of the human cognitive map (O’Keefe and Nadel, 1978), or internal representation of an environment. Support for this conceptualization has come from correlational research relating hippocampal volume to navigational experience and use of spatial strategies. Hippocampal volume was significantly correlated with time spent as a London taxi driver (Maguire et al., 2000) but not with time spent as a London bus driver (Maguire et al., 2006), suggesting that navigational experience—not route following—contributed to hippocampal size. London taxi drivers also had larger posterior hippocampi than controls, while controls had larger anterior hippocampal volume, implicating the posterior hippocampi as storing spatial representations (Maguire et al., 2000). Furthermore, number of years of navigation experience driving taxis was associated with increasing posterior and decreasing anterior hippocampal volume (Maguire et al., 2006), but navigational expertise in non-taxi drivers was not (Maguire et al., 2003). Thus, experience using one’s spatial representations, rather than “innate” ability, seems to drive changes in the hippocampus (Maguire et al., 2003). Hippocampal gray matter is also related to strategy use in a virtual radial maze task in both young (Bohbot et al., 2007) and older adults (Konishi and Bohbot, 2013) such that those with more hippocampal gray matter are more likely to use navigation strategies that depend on a cognitive map. Moreover, after learning the layout of a real-world environment from direct experience, right posterior hippocampus volume was positively correlated with pointing to various locations in the environment from imagined locations and headings (Schinazi et al., 2013).

Numerous brain regions have been implicated in allocentric coding, such as the retrosplenial cortex with its surrounding areas and the hippocampus with its surrounding areas, and these areas likely interact to support spatial activities (Ekstrom et al., 2014). The retrosplenial cortex-posterior cingulate (RSC/PC) region and presubiculum have been implicated in orienting to the larger environment and might translate between the egocentric coding from the parietal lobe and the allocentric coding in the medial temporal lobes (Epstein, 2008). The RSC does this by anchoring spatial representations of location and facing direction to local topological features (Marchette et al., 2014). This can be seen in individuals with damage in the RSC as they are unable to use familiar landmarks to provide them with a sense of orientation to the larger environment (Maguire, 2001). In a repetition suppression study, headings that faced the same direction were more suppressed in the RSC/PC than headings that faced different directions (Baumann and Mattingley, 2010), demonstrating that allocentric directions are coded in the RSC. Using multivoxel pattern analysis, the RSC, along with the left presubiculum and parietal-occipital sulcus, was found to code location identity, while the right presubiculum coded facing direction relative to the cardinal directions (Vass and Epstein, 2013).

To summarize, head-direction cells code allocentric headings in rats. In humans, hippocampal volume has been associated with environmental-scale spatial learning, skills, and experience, along with strategy use; whereas, the RSC/PC region and presubiculum are likely involved in orientation and the coding of facing direction.

### Heading Recall Task

**FIGURE 1:**
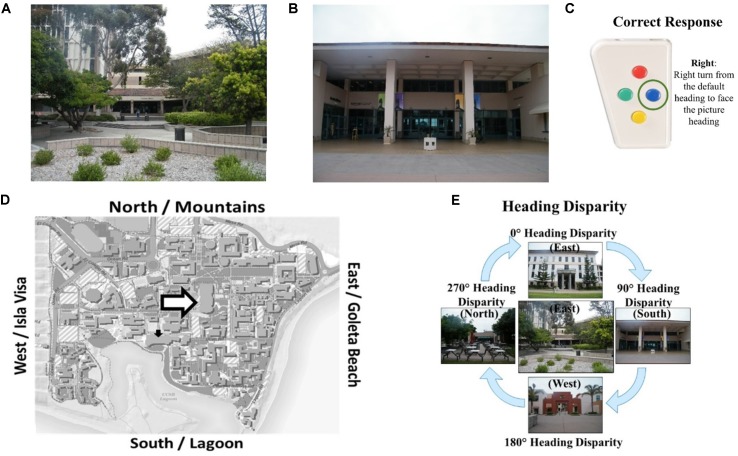
The Heading Recall task: **(A)** the view out the window from the experiment room while the participant faces east (i.e., default heading is east), **(B)** a photograph of campus is presented (i.e., picture heading facing south), **(C)** the correct response, and **(D)** the default heading (white arrow) and picture heading (black arrow) on a campus map. Calculating heading disparity **(E)**: when facing east (center), an east-facing picture (top) will have a heading disparity of 0°, but a south-facing picture (right) will have a heading disparity of 90°.

While head-direction cells have yet to be identified in humans, the Heading Recall task (or what we have previously called the “Allocentric-Heading Recall task”; Burte and Hegarty, 2012, 2013, 2014) was designed as a method for investigating the functioning of a possible head-direction system in humans (Sholl et al., 2006). In the Heading Recall task, participants are placed in an initial physical facing direction (called a “default heading,” Figure 1A). The second facing direction (called a “picture heading,” Figure 1B) is a photograph of a building from a familiar environment. For example, a participant might be seated facing east—the “default heading”—and see a photograph of a bookstore taken while the photographer was facing south—the “picture heading.” The participant should respond by turning toward the right (a turning response was used in Sholl et al., 2006; Burte and Hegarty, 2012) or pressing the right button (a button-press response was used in Burte and Hegarty, 2013), to indicate that when starting facing east one would need to turn right to face south (Figures 1C,D). The relationship between the default and picture headings is called “heading disparity” (Figure 1E). When the headings face the same direction, heading disparity equals 0°. Heading disparity is 180° when headings are facing opposite directions. Heading disparity is 90° (or 270°) when the headings are to the right (or left) of one another. It is important to note that while the Heading Recall task is most easily described in text using cardinal directions, cardinal directions are never used in the task or instructions.

Sholl et al. (2006) and colleagues hypothesized that comparing two headings that faced the same direction would be quick and accurate, because the firing of putative human head-direction cells in response to the participant’s physical facing direction would prime the firing of head-direction cells in response to the picture heading. Conversely, comparing headings that faced opposite directions would be slow and inaccurate as the firing of head-direction cells from the participant’s physical facing direction would inhibit the heading response from the second heading. This is due to the suppression effect of cells that code headings that are antipodal to the heading that is currently activated (Sharp et al., 2001). Consistent with these predictions, Sholl et al. (2006) found that accuracy decreased with heading disparity from 0° compared to 180°, and found a similar but marginal trend for increased correct decision latencies (i.e., response times for correct trials) with heading disparity (Experiment 1, Sholl et al., 2006). This finding provided support for their hypothesis that head-direction signals in humans function using an attractor network, similar to animal models. This alignment effect is similar to other alignment effects found relative to the body, such as the sensorimotor alignment effect (e.g., Kelly et al., 2007), and memory alignment effect (e.g., Shelton and McNamara, 1997, 2001).

In addition, Sholl et al. (2006) found that accuracy and correct decision latencies were correlated with self-assessed sense-of-direction, but were not correlated with distance to photographed location. These findings were interpreted as evidence that people’s conceptualization of their own sense-of-direction is reflective of how well their head-direction cells code and compare headings, and that participants likely did not use a “mental walk” strategy to compare headings (an alternative hypothesis to their attractor network hypothesis). A study conducted in the environment used in the present study replicated these results (Burte and Hegarty, 2012) and found that familiarity ratings were related to self-assessed sense-of-direction and accuracy. Participants with a better sense-of-direction tend to be more familiar with locations in the environment and, not surprisingly, familiarity predicted accuracy in comparing headings as recognizing the pictured location is essential to the comparison. A follow-up study, in which the response mode was changed from turning in a chair to a button-press (Burte and Hegarty, 2013), revealed similar results along with a gender difference in performance: males were more accurate than females. This study also revealed that participants can accurately respond to the Heading Recall task using egocentric (e.g., right, left, front, back) or allocentric (e.g., cardinal directions, large-scale spatial referents) frames of reference, although allocentric frames of reference tend to result in higher accuracy rates (Burte and Hegarty, 2013).

### Relative Heading Task

The Relative Heading task, used in the present research, was designed to investigate the nature of the alignment effect found in the Heading Recall task (Burte and Hegarty, 2014). In designing this new task, we also identified and corrected a common error made by participants^1^ and created a task that could be administered in an MRI scanner.

The Relative Heading task was designed to test whether the alignment effect found in the Heading Recall task (or “original alignment effect”) was a sensorimotor effect caused by an attractor network such as the head-direction cells in animals or whether it could be due to other computational difficulty in comparing headings. Comparing headings that are facing the same direction would likely be computationally easier than comparing headings that are facing opposite directions. This computational difficulty would be present regardless of whether the participant’s physical facing direction was priming or suppressing the firing of head direction cells in response to the picture heading, or not. The computational difficulty of comparing heading could result in an alignment effect that was similar to the one Sholl et al. (2006) found. To disentangle sensorimotor effects (due to a head direction system) from the computational difficulty of comparing headings, we designed the Relative Heading task in which the “default heading” is an imagined heading rather than the participant’s physical heading. If we found an alignment effect in the Relative Heading task, then it suggests that the alignment is due to computational difficulty in comparing headings, and not just a sensorimotor effect caused by a hypothesized head direction system in humans.

**FIGURE 2:**
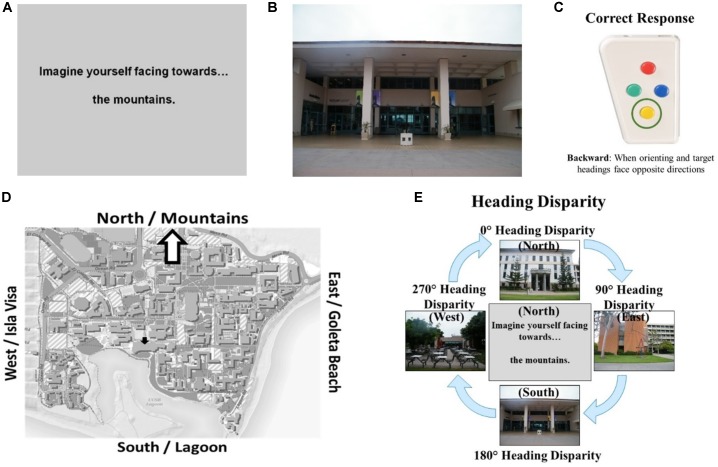
The Relative Heading task: **(A)** an orienting heading facing the mountains (i.e., north), **(B)** a photograph of campus facing the lagoon (i.e., a target heading facing south), **(C)** the correct response, and **(D)** the orienting heading (white arrow) and target heading (black arrow) on a campus map. Calculating heading disparity **(E)**: when facing north (center), a north-facing picture (top) will have a heading disparity of 0°, but a south-facing picture (bottom) will have a heading disparity of 180°.

The Relative Heading and Heading Recall tasks differ in how the initial “default” facing direction is presented. In the Relative Heading task (Burte and Hegarty, 2014), the initial heading is an imagined orientation presented in text (called an “orienting heading,” Figure 2A) in contrast with the Heading Recall task, in which it is the participant’s current physical facing direction. In both cases, a pictured facing direction presented by a photograph of a building from a familiar environment or the “target heading” (see, Figure 2B). For example, a participant is presented with an orienting heading telling them to imagine facing the mountains (the mountains are north of their location) and then is presented with a photograph of the bookstore that was taken by a photographer facing south (Figure 2D). The participant should respond with pressing the backward button, to indicate the difference between the two headings (Figure 2C). The heading disparity is the difference between the orienting and target heading (Figure 2E). Again, while the Relative Heading task is most easily described using cardinal directions, cardinal directions are never used in the task or instructions, so participants do not need to know the relationship between the facing directions and the cardinal directions to answer correctly, as they can use egocentric and/or allocentric frames of reference to compare the headings.

A previous study of the Relative Heading task found a partial alignment effect for correct decision latencies (180° was slower than 90°), but no alignment effect for accuracy. These results were interpreted as indicating that when the participant’s physical facing direction is taken out of the task, the attractor network cannot prime or suppress the head-direction cell response to the target heading. Previous works has also found that when participants learned an environment through direct experience (as is the case in the present studies), and their physical facing direction was not part of the heading comparison, their performance did not show an alignment effect (Presson and Hazelrigg, 1984). As in previous research on the Heading Recall task, performance in the Relative Heading varied widely across individuals, and participants with better self-assessed sense-of-direction were more accurate.

### Current Study

In this paper, our first objective was to investigate the factors that contribute to variation in performance of the Relative Heading task. We investigated the effects of previously mentioned predictors of Relative Heading performance: heading disparity (0°, 90°/270°, and 180°), self-assessed sense-of-direction, distance, familiarity, and gender. Our predictions follow previous findings: at most a partial alignment effect, better performance for those with better self-assessed sense-of-direction, no relationship with distance, better performance on high-familiarity pictures, and better performance for males than females. For familiarity, we used three measures: subjective familiarity rating, correctly naming the building in a photograph, and correctly identifying the nearest neighboring building. These measures allowed us to investigate effects of objective measures of familiarity (i.e., naming and nearest building) and not just self-reported ratings. Since other environmental-scale spatial tasks, such as wayfinding, spatial orientation, and pointing tasks, are all impacted by environmental familiarity (O’Neill, 1992; Prestopnik and Roskos–Ewoldsen, 2000; Nori and Piccardi, 2011), we predict that the ability to determine the facing direction of a photograph will also depend on environmental familiarity.

We separated effects that occur on the participant-level (i.e., averaged over photographs/trials), reflecting individual differences in directional sense, and effects that occur on the trial-level (i.e., for each trial nested under each participant) in separate models. Using linear mixed models, the impact of familiarity, distance, and direction toward the photographed location on performance for each photograph could be modeled. Since participants were oriented to the testing location (during brain scanning), distance and direction from the testing location to the pictured location were included to evaluate whether the participant’s physical location and orientation in the environment (which is separate from the target and picture headings) impacted their task performance. While previous work did not find a relationship between distance (from one’s physical location to the location in the picture) and performance (Sholl et al., 2006; Burte and Hegarty, 2012), those analyses were completed using correlations of data aggregated across trials; we have included them in the current study so the effects of distance could be investigated on a trial-by-trial basis using linear mixed modeling. Since participants were oriented to the environment while in the scanner, we tested whether the directional relationship between their bodies and the target locations impacted performance, as this has not been previously investigated.

#### Structural Differences

The second objective of this study was to examine whether differences in directional sense are related to structural differences in the brains of individuals, and specifically whether variation in directional sense and self-assessed sense-of-direction were related to hippocampal volume. To accomplish this, we identified brain areas that showed a relationship between volume and both performance on the Relative Heading task and self-reported sense-of-direction. Given that hippocampal volume has been associated with environmental-scale spatial learning, skills, and experience (e.g., Maguire et al., 2000, 2003, 2006), we predict that hippocampal volume would also be related to self-reported sense-of-direction because self-reported sense-of-direction is highly predictive of environmental-scale spatial abilities (e.g., Kozlowski and Bryant, 1977; Hegarty et al., 2002; Hegarty et al., 2006). Since we have proposed that directional sense underlies human navigational abilities, we also predict that hippocampal volume will be related to Relative Heading performance.

#### Functional Differences

The third objective of this paper was to investigate the neural basis of directional sense and its variability. Given the steps needed to complete the Relative Heading task, we predict that brain areas involved in the following processes are likely to show task-relevant activation: (1) imagining the orienting heading (specified in text), (2) visually identifying the pictured location, (3) identifying the target heading, and (4) comparing the two allocentric headings.

##### Identifying allocentric headings

The first and third steps in the Relative Heading task require identifying allocentric headings from the text indicating the orienting heading and from the photograph indicating the target heading, respectively. Since the RSC/PC region and presubiculum are likely involved in orientation and the coding of facing directions (e.g., Epstein, 2008; Vass and Epstein, 2013; Ekstrom et al., 2014), these regions might show activation as participants are identifying facing directions using the imagined and visual landmarks provided by the orienting and target headings, respectively.

There is evidence that the parahippocampus, instead of the hippocampus, may be more involved in identifying the headings in the Relative Heading task. This is because the hippocampus responds to specific spatial locations, whereas the parahippocampal region responds to views of landmarks. Using intracranial electrodes while participants completed a virtual navigation task, the place-responsive cells were found in the hippocampus and location-independent view-responsive cells were found in the parahippocampus (Ekstrom et al., 2003). The parahippocampal cortex was also found to be more responsive to landmark recognition and associations with spatial locations (Ekstrom and Bookheimer, 2007). More specifically, the parahippocampus seems to be focused on processing the visual-spatial structure of scenes (Zhang et al., 2012).

In sum, the retrosplenial cortex with its surrounding areas and the hippocampus with its surrounding areas support slightly different spatial information. These differences suggest that the RSC/PC will likely be involved in the comparison of allocentric-headings in the Relative Heading task. When these areas were compared directly, the retrosplenial cortex was more involved in orientation changes and the hippocampus was more involved in self-motion changes (i.e., motion with orientation changes; Gomez et al., 2014). Since the Relative Heading Task involves solely orientation changes, we predict that the retrosplenial cortex will show greater activation as the heading disparity (or orientation change) increases.

##### Visually identifying the pictured locations

The second process in completing the Relative Heading task is to visually identify the pictured target heading. This process might be intertwined with the first and third processes as participants might imagine themselves in the environment facing the large-scale referent given in the orienting heading, and/or imagine themselves taking the photographer’s perspective for the target heading. Imagining being in the environment (i.e., an egocentric perspective) or imagining a map or an aerial view of the environment (i.e., an allocentric perspective) might activate visual areas and areas associated with memory for locations.

Since one way of completing the Relative Heading task is by imagining taking the photographer’s location and heading in the environment, areas that support taking an egocentric perspective are likely to become active. The right inferior parietal and bilateral medial parietal areas have been associated with supporting egocentric movement through a virtual town (Maguire et al., 1998), as opposed to right hippocampus and caudate that were associated with knowing where a place is located and navigating to that place quickly and accurately. Studies involving navigation have also found activation in the frontal and parietal lobes (e.g., Grön et al., 2000; Iaria et al., 2003), implicating these areas in spatial decision making and in coordinating egocentric movement through an environment.

##### Comparing allocentric headings

Given that the RSC/PC acts as something of a mediator between the parietal lobe and the medial temporal lobe, it has been proposed that this area translates between the egocentric coding of the parietal lobe and the allocentric coding of the medial temporal lobe (Byrne et al., 2007; Epstein, 2008). A study by Lambrey et al. (2012) supported this translational hypothesis, with the researchers finding that the RSC/PC was involved in updating imagined self-rotations. These imagined self-rotations required the updating of one’s egocentric location within an allocentric reference frame, which is similar to the process of comparing allocentric headings in the Relative Heading task. This suggests that the RSC/PC might be involved not only in coding the allocentric headings of the orienting and target headings, but also in comparing allocentric headings.

## Materials and Methods

### Ethics Statement

This study was carried out in accordance with the recommendations of the Human Subjects Guidelines and Procedures, from the University of California Santa Barbara’s Office of Research. The protocol was approved by the Human Subjects Committee. All subjects gave written informed consent in accordance with the Declaration of Helsinki. Participants completed consent forms before and were debriefed after both the prescreening and experiment.

### Prescreening

Since familiarity with the campus was essential to completing the Relative Heading task, we used a pre-screening process to select participants who had spent at least a year on campus, and who were highly familiar with the photographed locations. Given the individual and gender differences previously found in the Relative Heading and Heading Recall tasks, we selected participants who represented a wide range of self-assessed sense-of-direction, and an equal number of males and females. We also selected participants who met the requirements for participating in an fMRI study.

#### Participants

Graduate and undergraduate students and staff from University of California, Santa Barbara (UCSB) completed the prescreening and were paid $20 (*N* = 104; female *N* = 59; male *N* = 45; aged 18–20 *N* = 64; aged 21–23 *N* = 33; aged 24+ *N* = 7; maximum age = 35).

#### Materials and Procedure

Participants completed demographics questions (age, gender, time spent on campus), a commonly used measure of self-assessed sense-of-direction—the Santa Barbara Sense of Direction (SBSOD) scale (Hegarty et al., 2002)— three familiarity assessments, and an fMRI screening questionnaire (native language, handedness, claustrophobic, metal screening, normal or corrected-to-normal vision). For the familiarity assessments, participants rated their familiarity with campus photographs on a 7-point rating scale (1 = “Very familiar” through 7 = “Not at all familiar”), selected the name of the photographed building (4-option multiple-choice), and selected the nearest building to the photographed building (4-option multiple-choice). Table 1 contains means and standard deviations for SBSOD scores and familiarity measures.

**Table 1:** Means, standard deviations, and *t*-tests for gender differences for prescreening participants.

	Prescreening (*N* = 104)		Females (*N* = 59)		Gender Difference	Males (*N* = 45)	
				
	*M*	*SD*	*M*	*SD*	*p*	*M*	*SD*
SBSOD 1 – poor SOD; 7 – good SOD	4.8	1.0	4.4	0.8	0.000	5.3	0.9
Familiarity Rating 1 – Very; 7 – Not familiar	1.7	0.9	1.9	1.0	0.04	1.5	0.6
Building Name Mean accuracy	94%	4%	94%	4%	0.24	95%	4%
Nearest Building Mean accuracy	85%	9%	85%	9%	0.72	86%	9%


#### Selection of Experimental Participants

Participants were selected for the fMRI experiment if they met these requirements: (1) at least 1 year of experience on the UCSB campus; (2) native English speaker; (3) right-handed; (4) not claustrophobic; (5) passed metal screening for fMRI; (6) normal or corrected-to-normal vision; and (7) high familiarity with the campus photographs. High familiarity was operationalized as a mean familiarity rating of 1.0–3.0 on the 7-point familiarity scale, correctly identifying at least 85% of photographed buildings, and correctly identifying at least 65% of buildings near the photographed buildings. Based on these criteria, 76 participants (73%) were eligible to participate.

Males in the eligible group rated their sense-of-direction as better on the SBSOD than females, *t*(102) = -5.48, *p* < 0.001. While males reported higher levels of familiarity with campus photographs, *t*(102) = 2.07, *p* < 0.05, they did not differ from females in objective measures of familiarity, that is, building name accuracy, *t*(102) = -1.18, *p* = 0.24, or nearest building accuracy, *t*(102) = -0.37, *p* = 0.72 (Table 1). In addition to the criteria reported in the previous paragraph, participants were selected to participate in the fMRI experiment based on their sense-of-direction relative to others of the same gender (such that the distribution of sense-of-direction within the fMRI participants approximated the distribution of sense-of-direction within all the prescreening participants of each gender). We invited this group of seventy-six participants to participate; however, not all those who were invited actually participated.

### Experiment

#### Participants

Fifty-six right-handed (female *N* = 27; male *N* = 29; aged 18–20 *N* = 36; aged 21–23 *N* = 17; aged 24+ *N* = 3; maximum age = 35) students and staff at UCSB gave informed consent as approved by the Institutional Review Board, completed the fMRI experiment, and were paid $50. Due to excess motion or technical difficulties with the response pad, 11 participants were excluded from the analysis, leaving the remaining forty-five participants (female *N* = 23; male *N* = 22; aged 18–20 *N* = 27; aged 21–23 *N* = 15; aged 24+ *N* = 3), for inclusion in the behavioral, structural, and functional analyses.

#### Stimuli

Both the practice tasks and Relative Heading task used two types of stimuli: orienting and target headings. The practice tasks used four photographs from within the experiment room (which faced the cardinal directions) for orienting headings and used either four photographs from within the experiment room or four photographed buildings (different buildings from those used in the main experimental task). The practice tasks used stimuli and headings that were similar to those used in the Relative Heading task so that participants could be introduced gradually to comparing headings.

**FIGURE 3:**
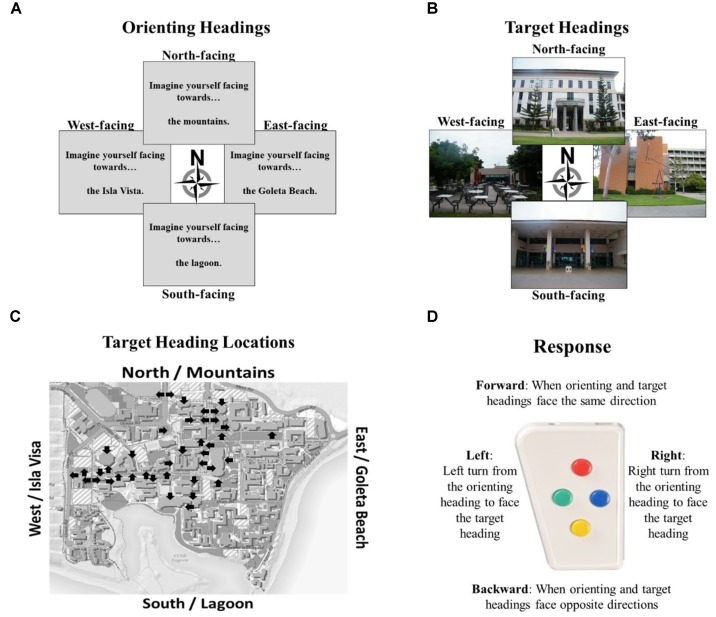
Components of the Relative Heading Task: **(A)** orienting headings as described by large-scale spatial referents from the UCSB campus, **(B)** target headings given by photographs facing familiar UCSB buildings, **(C)** locations and facing directions of all photographed buildings, and **(D)** button box used to respond with the directional relationship starting from the orienting heading towards the target heading.

The Relative Heading task stimuli included four orienting headings specified in text using commonly used large-scale landmarks to indicate orientation (e.g., “Imagine facing toward the mountains/Goleta Beach/lagoon/Isla Vista”; Figure 3A), and forty target headings that consisted of photographs of prominent UCSB buildings (Figure 3B). The photographs of buildings were taken on a cloudy day to avoid directional cues from the sun, were cropped to exclude views of other buildings or landmarks to avoid directional cues beyond the building itself, and faced the cardinal directions (10/direction; Figure 3C). These photographs were sourced from the most familiar photographs used within the Relative Heading task (Burte and Hegarty, 2014), as UCSB students and staff had already demonstrated familiarity with these locations. Experiment participants (i.e., those who completed the experiment in the fMRI scanner) were all highly familiar with these photographs (mean familiarity rating of 1.7/7, mean correct building naming was 94%, and mean correct near building naming was 83%).

The orienting and target headings were aligned with the cardinal directions, the overall layout of campus, and the experiment room in which the participants completed the practice tasks, and the MRI scanner room in which the participants completed the Relative Heading task. This alignment makes the task simpler, as accuracy dramatically drops when using photographs that are misaligned to the campus layout (Experiment 3, Sholl et al., 2006). In the task instructions and orienting procedures, cardinal directions were never mentioned because they are not needed to complete the task. While participants do not need to be oriented to the environment to complete the Relative Heading task (disorientation does not impact performance compared to being oriented; Burte and Hegarty, 2014), we wanted to ensure that all participants were similarly oriented so we checked that participants knew how the experiment room and MRI scanner room were aligned with the orienting and target headings. Neither room had windows so participants did not have visual access to the environment.

#### Behavioral Paradigm

##### Practice tasks

The experiment started with three practice tasks, completed in an experiment room outside of the scanner, to illustrate what allocentric headings are and how to compare them. Participants were oriented to the campus environment, so they knew how directions in the experiment room related to the campus. The first practice task was the Heading Recall task, which used the participant’s physical facing direction as the default heading and photographs taken within the experiment room as picture headings (8 trials). The second practice task was a simplified version of the Relative Heading task, in which participants were given orienting headings via text (e.g., “Imagine facing toward the mountains.”) but this simplified version used photographs taken within the experiment room as target headings (8 trials). The third practice task was the Relative Heading task, in which orienting headings were given via text and target headings were given via photographs of buildings on the UCSB campus (12 trials). During this task, participants practiced responding in less than 5 s, as that was the time limit they would have for responding when in the scanner. Participants were informed that they would perform the third practice task in the scanner, except with different photographed buildings for target headings.

##### Relative heading task in fMRI

After the practice tasks, participants were taken to the Brain Imaging Center. Once there, they pointed toward the orienting headings to reorient them to the outside environment (to ensure that all participants had a similar level of orientation to the campus), completed three anatomical scans (localizer, T1 MP-RAGE, and GRE), and completed the Relative Heading task during functional scanning.

For the Relative Heading task, the 40 campus photographs were split into two sets, so that the first four functional runs used half of the target heading photographs, and the second four functional runs used the other half. The photographs from each heading were split randomly between the two sets (e.g., 5 east-facing in each set), but the order of the sets was not counterbalanced, to allow for similarity analyses across the sets (not reported in this manuscript).

On each trial, participants were first given an orienting heading (e.g., “Imagine facing toward the mountains”), and then were shown a campus photograph (e.g., photographer was facing east to photograph the entrance to the library). Their task was to indicate the heading of the campus photograph relative to the orienting heading. In this example, they should press the right button because facing toward the mountains (i.e., north) one would need to turn to the right to face that view of the library (i.e., east). Participants responded to the Relative Heading task using a four-directional response pad (Figure 3D): (1) the direction toward the participant’s feet, or “forward,” which indicated that the orienting and target heading faced the same direction; (2) the direction toward the participant’s right, which indicated that the target heading was 90° to the right (clockwise) of the orienting heading; (3) the direction toward the participant’s head, or “backward,” which indicated that the orienting and target headings were 180° apart; and (4) the direction toward the participant’s right, which indicated that the target heading was 90° to the left (counterclockwise) of the orienting heading. For each trial, we calculated accuracy and decision latency (i.e., time from viewing the target heading until a response was given).

#### Imaging

##### Imaging procedures

Imaging was performed in the Brain Imaging Center at UCSB using a 3T Siemens Trio MRI scanner, which was equipped with high-performance gradients. Stimulus presentation was controlled by an ASUS A55A laptop using PsychToolbox for Matlab^2^. The stimuli were presented using an LCD projector that back-projected the images onto a screen at the back of the bore, and was viewed using a mirror mounted to the head coil. Within the head coil, foam padding was used for head stabilization. Participants responded using a 4-button magnet-compatible fiber-optic button box that communicated directly with the laptop and PsychToolbox.

First, a high-resolution T1-weighted structural image was acquired (MP-RAGE: TR = 1700 ms, TE = 2.97 ms, RF flip angle = 9°, bandwidth = 240 Hz, voxel size = 1.0 mm × 1.0 mm × 1.1 mm), and then gradient-recalled echo-planar imaging was used to acquire the functional images (TR = 2000 ms, TE = 30 ms, RF flip angle = 90°, gradient-echo pulse sequence, 33 contiguous axial slices at 3.0 mm thick with a 0.5 mm slice gap, and an in-plane resolution of 64 × 64 pixels within a 192 cm field of view, producing voxels of 3mm × 3mm × 3mm). The experiment employed an event-related design and consisted of eight 7-min functional scans of the Relative Heading task. Each functional scan was preceded by five volumes to approach steady-state magnetization, which were discarded.

**FIGURE 4:**
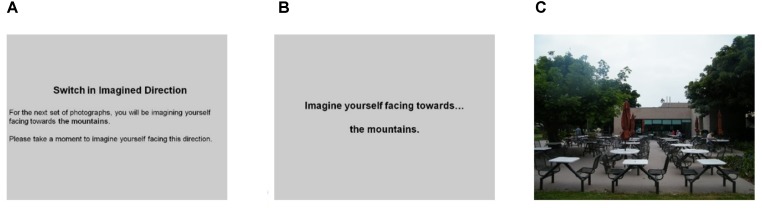
Mini-block order: **(A)** notification, **(B)** orienting heading followed by jitter, and **(C)** target heading followed by jitter. Repeat same orienting heading with different target headings for 6–8 times before switching orienting heading with a new notification.

The functional scans consisted of mini-blocks that allowed participants to keep returning to the same orienting heading for a series of trials before switching orienting heading. This design was used because some participants experienced motion sickness when the orienting and target heading changed every trial. The mini-blocks were presented in the following manner: (1) mini-block notification slide “For the next trials, you will be imagining facing toward the [mountains/Goleta Beach/lagoon/Isla Vista]” for 4 s; (2) orienting heading for 3 s; (3) average 1 s jitter with blank screen; (4) target heading for 5 s, during which time participants responded; (5) average 1 s jitter with blank screen; and (6) repeat steps 2–5 for 4–8 target heading stimuli (Figure 4). Mini-blocks were arranged so that the same orienting heading was not repeated back-to-back, with runs containing 6 mini-blocks. The order of the mini-blocks, and the order of the target headings were arranged in a non-predictable quasi-random fashion. This design ensured that each unique combination (orienting heading with one of the 40 target headings) was repeated at least twice for every participant, for a total of 281 trials across all eight functional runs. Since participants had a 5 s period to respond, trials on which they did not respond within that time frame were counted as incorrect trials. Accuracy along with decision latencies on correct trials were calculated.

##### Structural preprocessing and analysis

Using each participant’s high-resolution T1-weighted structural scan, FreeSurfer’s^3^ fully automated cortical surface-based pipeline (Dale et al., 1999; Fischl et al., 1999) applies a Talairach registration procedure using the MNI305 atlas, intensity normalization, skull-stripping, white matter labeling and segmentation, the intensity normalization to reveal the pial surface, pial surface labeling and segmentation, and the white and pial surfaces are overlaid on the original T1 to calculate cortical thickness. Using FreeSurfer’s fully automated volume-based pipeline (Fischl et al., 2002, 2004), the T1-weighted structural scan undergoes registration to the MNI305 atlas, initial volumetric labeling, intensity normalization, volumetric alignment is completed using a high dimensional non-linear alignment to the MNI305 atlas, and volume labeling.

To know how variation in cortical volume was related to sense-of-direction and ability to compare headings, a whole-brain surface-based group analysis was performed on volumetric data (in mm^3^) that was registered to FreeSurfer’s surface atlas (fsaverage) using spherical cortical registration. Surface smoothing using FWHM 10 mm was run. A general linear model (GLM) tested correlations between cortical volume with Relative Heading task performance (mean accuracy and correct decision latencies over all trials) and SBSOD scores. To correct for multiple comparisons, a False Discovery Rate (FDR) of 0.05 was used.

To understand how variation in subcortical volume was related to sense-of-direction and ability to compare headings, a regional analysis was completed using the Desikan/Killiany Atlas (Desikan et al., 2006) that was registered using fsaverage so that corrections for total brain volume are not needed. A GLM tested for correlations between subcortical volume with Relative Heading task performance and SBSOD scores. FreeSurfer’s structural region-of-interest analysis includes 33 subcortical areas but does not include corrections for multiple comparisons.

Given our interest in hippocampal size, we used FreeSurfer’s automated segmentation of the hippocampal subfields (Iglesias et al., 2015) to calculate the volume of the four parts of the hippocampus (CA1, CA2/3, CA4, and tail) and surrounding areas (parasubiculum, presubiculum, and subiculum). Since only uncorrected volumes were produced, we corrected the volumes using total intracranial volume (eTIV). Using a multilevel linear model, we investigated the relationship between hippocampal subregions and SBSOD scores.

##### Functional preprocessing and analysis

FreeSurfer’s FS-FAST preprocessing and analysis stream was used. Preprocessing included motion correction, slice-timing correction, B0 distortion correction, spatial normalization, and spatial smoothing (5 mm FWHM). The group analyses included registration of fMRI scans to the anatomical space, registering the anatomical to MNI305 and the surface atlas (fsaverage), registering fMRI scans to MNI305 and fsaverage, and merging subjects using fsaverage.

To investigate the neural processes that support the comparison of headings, we set up the functional analyses to focus on how heading disparity corresponds with BOLD amplitude during the target heading TRs. We focused our analyses on target heading TRs because that was the time in which participants were making the comparison (as opposed to orienting heading TRs where participants knew only the starting direction from which they would subsequently be making comparisons). Given our focus on understanding heading comparison, our analyses focused on heading disparities (i.e., the relationship between orienting and target headings). Specifically, these analyses focused on identifying brain areas in which their functional activity reflected a linear relationship as a function of heading disparity. If a functional area was involved in the comparison of headings, then activity in that area should show a positive or negative linear relationship with heading disparity. To identify these areas, we used parametric modulation analysis.

The parametric modulation analysis required selecting target heading TRs and assigning a heading disparity to each target heading TRs. Using paradigm files for each run completed by each participant, an offset and a slope were specified. It was the slope parameter that investigated whether the amplitude of the predicted hemodynamic response was modulated based on the heading disparity: (0) TRs other than target heading TRs; (1) target headings TRs with 0° heading disparity; (2) target headings TRs with 90° or 270° heading disparity; and (3) target headings TRs with 180° heading disparity.

The first-level GLM was specified with an event-related design, SPM’s canonical HRF (hemodynamic response function) model with 0 derivatives^4^, 2nd order polynomials for nuisance drift modeling, and motion correction parameters as nuisance regressors. The resulting group maps of the t statistics were computed using bidirectional contrasts.

In the higher-level GLMs, the offset and slope parameters were both modeled using a simple [1 0] contrast. A volume-based correction for multiple comparisons was applied, with a voxel-wise threshold of *p* < 0.001 and a cluster-wise threshold of *p* < 0.05. Clusters were assigned labels using the MNI 305 atlas, and FreeSurfer’s cortical and subcortical atlases.

## Results

### Behavioral Results

#### Individual, Gender, and Familiarity Differences

First, we investigated whether previous findings of large individual differences and gender differences in heading comparison tasks (Sholl et al., 2006; Burte and Hegarty, 2012, 2013, 2014) were replicated in the current study. In line with those findings, large individual differences were found in accuracy (range = 25–89%) and decision latency for correct trials (range = 1.7–3.2 s). Males were more accurate, *t*(43) = -3.63, *p* < 0.01, left fewer trials unanswered, *t*(43) = 3.34, *p* < 0.01, took less time to respond correctly, *t*(43) = 2.25, *p* < 0.05, and reported having a better sense-of-direction than females, *t*(43) = -3.69, *p* < 0.01 (Table 2). There were no gender differences in familiarity. In terms of the relationship between sense-of-direction and directional sense, SBSOD scores were significantly correlated with accuracy, *r*(43) = 0.41, *p* < 0.01, and correct decision latency, *r*(43) = -0.36, *p* < 0.05.

**Table 2:** Means, standard deviations, and t-tests for gender differences for fMRI participants.

	Experiment (*N* = 45)		Females (*N* = 23)		Gender Difference	Males (*N* = 22)	
				
	*M*	*SD*	*M*	*SD*	*p*	*M*	*SD*
Relative Heading Accuracy	71%	17%	63%	20%	0.001	80%	8%
Relative Heading Unanswered trials	9%	3%	11%	4%	0.002	8%	2%
Relative Heading Correct Decision Latency	2.4 s	0.4 s	2.5 s	0.5 s	0.03	2.2 s	0.3 s
SBSOD 1 – poor SOD; 7 – good SOD	4.9	1.0	4.5	0.8	0.001	5.4	0.9
Familiarity Rating 1 – Very; 7 – Not familiar	1.5	0.5	1.6	0.4	0.36	1.4	0.5
Building Name Mean accuracy	94%	4%	94%	4%	0.67	95%	5%
Nearest Building Mean accuracy	86%	9%	86%	9%	0.97	86%	9%


#### Regression Models

To understand how participant demographics and pre-screening measures predicted variation in performance on the Relative Heading task, we used stepwise linear regression models. The following predictors were included: SBSOD score, gender, familiarity rating, building naming accuracy, and nearest building accuracy.

**FIGURE 5:**
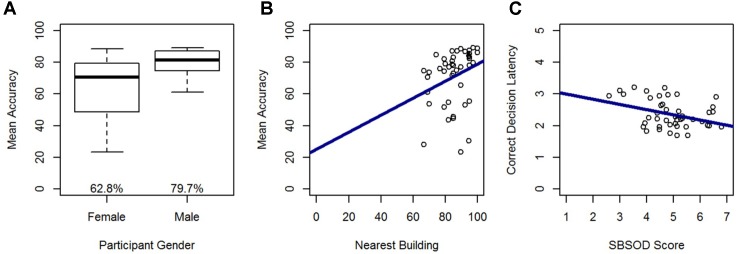
Relative Heading accuracy as predicted by **(A)** gender and **(B)** nearest building accuracy. Relative Heading correct decision latency as predicted by **(C)** SBSOD scores. For all box plots, the box center represents the median, the box top and bottom indicate the first and third quartile, the whiskers represent a 95% confidence interval, circles represent outliers, and mean values are provided. For all scatter plots, regression lines are in blue.

In a model predicting accuracy, gender (β = -0.49; *t* = -3.81, *p* < 0.001) and nearest building accuracy (β = 0.28; *t* = 2.21, *p* < 0.05) were significant predictors and explained 32% of the variance in accuracy, *F*(2,42) = 9.68, *p* < 0.001. Males out-performed females, and accuracy in the Relative Heading task increased with greater accuracy on the nearest building task (Figure 5). In a model predicting correct decision latency, score on the SBSOD (β = -0.36; *t* = -2.49, *p* < 0.05) was the sole significant predictor, explaining 11% of the variance in correct decision latency, *F*(1,43) = 6.20, *p* < 0.05. Correct decision latencies were shorter for those with a better sense-of-direction (Figure 5).

#### Linear Mixed Models

To identify the variables that impacted performance on each combination of orienting heading and photographed location (which included 2 trials per participant), we used the “lme4” package in R version 3.1.2 (Bates et al., 2015) to run linear mixed models. We ran a series of three models: (1) a null model that included random effects for each participant and orienting heading-photographed location combination; (2) a model that added fixed effects for familiarity rating (1–7), building naming accuracy (0,1), nearest building accuracy (0,1)^5^, gender (male = 1), SBSOD score (1–7), and blocks within each half of the experiment; and (3) a model that added fixed effects for orienting heading direction (N, E, S, W), target heading direction (N, E, S, W), heading disparity (0, 90, 180°), direction toward target (i.e., direction from the participant’s physical orientation in the scanner to photographed location), and distance from experiment location to target. Three models were used so that the explanatory power of variables (such as familiarity) that might impact performance, but were not of primary interest in this study (i.e., variables in Model 2), were accounted for before the explanatory power of the task-level variables of interest (i.e., variables in Model 3) was investigated. The models were compared using likelihood ratio chi-squares to determine if the fixed effects added predictive power (χ^2^). The estimates and standard errors for each fixed effect for each model appear in Table 3.

**Table 3:** Estimates and standard errors for linear mixed models.

Accuracy	*SS*	*F*	*p*	Decision latency	*SS*	*F*	*p*	Accuracy	*SS*	*F*	*p*	Decision latency	*SS*	*F*	*p*
Model 2				Model 2				Model 3				Model 3			
Familiarity rating	18.7	112.9	0.000	Familiarity rating	10.9	18.3	0.000	Target heading	2.5	5.0	0.003	Orienting heading	10.1	5.7	0.001
Half ^∗^ block	2.8	17.0	0.000	Half ^∗^ block	72.2	121.7	0.000	Signed direction	2.1	12.6	0.001	Signed direction	5.5	9.4	0.003
Nearest building	0.7	4.3	0.04									Heading disparity	24.8	41.9	0.000
Gender (Male = 1)	2.1	12.5	0.001												


Model 2 revealed that familiarity rating, nearest building accuracy, gender, and blocks within each half of the experiment were all significant predictors of accuracy, and this model significantly outperformed the random effects model (Model 1), χ^2^(4) = 148.29, *p* < 0.001. As expected, accuracy increased with greater familiarity ratings (nearer to 1), when participants could accurately identify the nearest building, and males were more accurate than females. Accuracy also increased from the first to the second half, indicating improvement with exposure to the task, and accuracy increased across blocks within each half of the trials, indicating improvements with exposure to the specific photographed locations used in each half.

**FIGURE 6:**
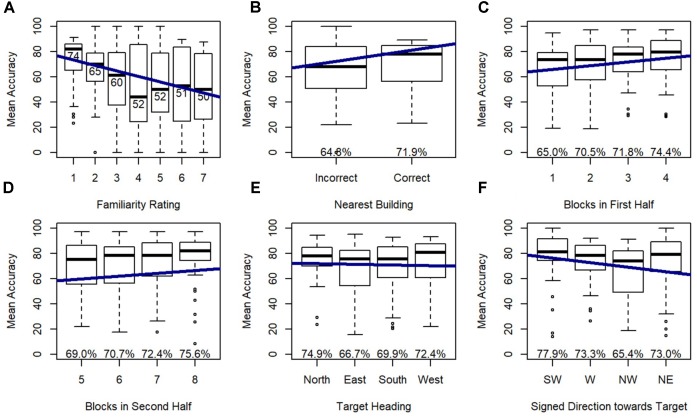
Relative Heading accuracy as predicted by **(A)** familiarity rating, **(B)** nearest building accuracy, **(C)** blocks in first half, **(D)** blocks in second half, **(E)** target heading, and **(F)** signed direction towards target: SW = –135 to –90, W = –90 to –45, NW = –45 to 0, NE = 0 to 45.

Model 3 indicated that target heading and signed direction toward target (i.e., direction from the participant’s physical orientation toward each photographed location) were significant predictors of accuracy, and this model significantly outperformed model 2, χ^2^(4) = 27.78, *p* < 0.001 (Figure 6) indicating that these effects added explanatory power above and beyond the explanatory power of familiarity, from Model 2. *Post hoc* tests revealed that participants were more accurate on north-facing than east-facing targets (*p* < 0.05) and south-facing targets (*p* < 0.001), and were more accurate on west-facing than south-facing targets (*p* < 0.05), replicating previous research with this environment. Accuracy dropped for targets that were north of the participant, which was toward the participant’s head while lying down.

#### Correct Decision Latency

Model 2 demonstrated that familiarity rating and blocks within each half of the experiment were significant predictors of correct decision latency, and this model significantly outperformed the random effects model (Model 1), χ^2^(2) = 140.96, *p* < 0.001. As expected, participants were faster to respond to familiar locations, and faster in the final block of each half of the experiment.

**FIGURE 7:**
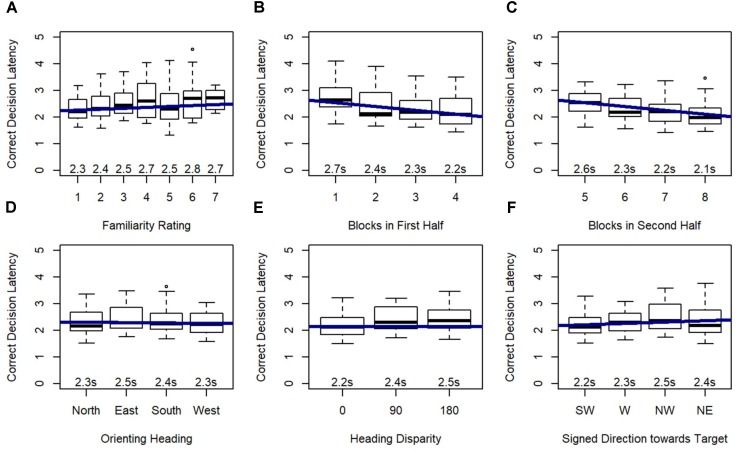
Relative Heading correct decision latency as predicted by **(A)** familiarity rating, **(B)** blocks in first half, **(C)** blocks in second half, **(D)** orienting heading, **(E)** heading disparity, and **(F)** signed direction towards target: SW = –135 to –90, W = –90 to –45, NW = –45 to 0, NE = 0 to 45.

Model 3 revealed that orienting heading, heading disparity, and signed direction toward target were significant predictors of correct decision latency, and this model significantly outperformed Model 2, χ^2^(5) = 58.60, *p* < 0.001 (Figure 7), again indicating that these effects added explanatory power beyond the explanatory power of familiarity (from Model 2). *Post hoc* tests revealed that participants responded faster on north-facing (*p* < 0.01) and west-facing (*p* < 0.001) than east-facing orienting headings, and faster on west-facing than south-facing orienting headings (*p* < 0.01). Critically, correct decision latency was faster when orienting and target heading matched (heading disparity was 0), and when the pictured location was closer to south or forward (i.e., closer to the participant’s feet).

### Structural Results

**FIGURE 8:**
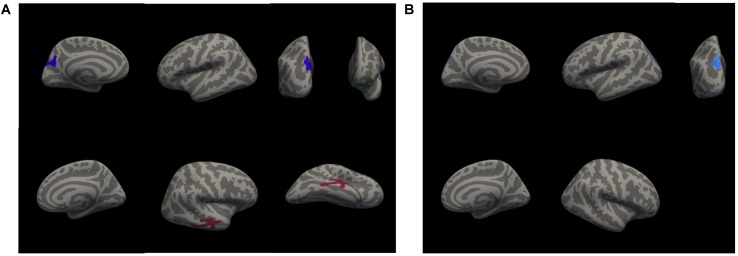
Clusters with significant positive correlations between cortical volume and accuracy **(A)**, and between cortical volume and correct decision latency **(B)**. Colors serve only to improve the visibility/distinguishability of clusters.

A whole-brain analysis was used to identify cortical volumetric variation associated with task performance and SBSOD scores (Figure 8 and Table 4). Task accuracy was positively correlated with the volume of the left lateral orbitofrontal (*mm*^3^ = 786, *p* = 0.004), left precuneus (*mm*^3^ = 1034, *p* = 0.0002) and right middle temporal gyrus (*mm*^3^ = 958, *p* = 0.0006). Correct decision latencies were positively correlated with the volume of the left superior parietal lobule (*mm*^3^ = 669, *p* = 0.01). No significant correlations were found for SBSOD scores.

**Table 4:** Areas showing significant positive correlations between volume with accuracy and correct decision latency.

Correlate	Maxima Coordinates (MNI)			Region name	Size (mm^3^)	*p*
				
	x	y	z			
Accuracy	-10.5	48.7	-21.8	Left lateral orbitofrontal	786	0.004
	-10.7	-66.4	32.0	Left precuneus	1034	0.0002
	57.9	-44.8	-13.3	Right middle temporal	958	0.0006
Correct decision latency	-18.4	-86.4	31.0	Left superior parietal	669	0.01


An ROI analysis was used to test for subcortical volumetric differences associated with task performance and SBSOD scores. The volume of the left ventral diencephalon (*p* = 0.008), left cerebellar white matter (*p* = 0.02), and right amygdala (*p* = 0.006) were significantly correlated with accuracy. No subcortical ROIs showed significant correlations with correct decision latencies. SBSOD scores were significantly correlated with both left (*p* = 0.006) and right (*p* = 0.02) hippocampal volume, such that participants with better sense-of-direction also had greater hippocampal volume. However, when we subdivided the hippocampus into its subregions (anterior, body, posterior, and tail), there was no significant relationship between hippocampal subregion volume and SBSOD scores. There was a significant relationship between volume in the right presubiculum and SBSOD scores (*p* = 0.005).

### Functional Results

**FIGURE 9:**
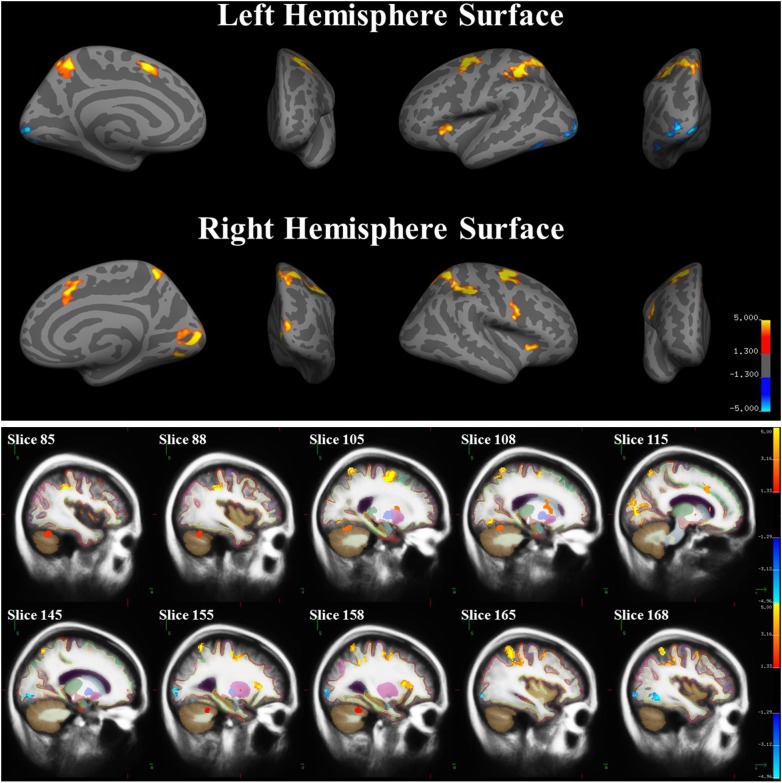
Brain areas with significant relationships between heading disparity magnitude and the hemodynamic response. Blue areas demonstrated a negative linear relationship (i.e., decreasing activity with larger disparities) and red/yellow areas demonstrated a positive linear relationship.

A whole-brain analysis was used to identify brain areas that exhibited a linear relationship between heading disparity magnitude and the hemodynamic response. This linear magnitude model found significant clusters of activation in the following areas: bilateral superior frontal gyrus (left 1: *mm*^3^ = 708, *p* = 0.0003; left 2: *mm*^3^ = 230, *p* = 0.0009; right 1: *mm*^3^ = 778, *p* = 0.0003; right 2: *mm*^3^ = 306, *p* = 0.0003), bilateral lateral occipital cortex (left 1: *mm*^3^ = 380, *p* = 0.0003; left 2: *mm*^3^ = 285, *p* = 0.0003; right: *mm*^3^ = 279, *p* = 0.0003), bilateral pericalcarine cortex (left: *mm*^3^ = 305, *p* = 0.0003; right *mm*^3^ = 704, *p* = 0.0003), left superior parietal lobule (*mm*^3^ = 1786, *p* = 0.0003), left fusiform gyrus (*mm*^3^ = 200, *p* = 0.001), right supramarginal gyrus (*mm*^3^ = 1594, *p* = 0.0003), right precentral gyrus (*mm*^3^ = 282, *p* = 0.0003), right lingual gyrus (*mm*^3^ = 251, *p* = 0.0003), right lateral orbitofrontal cortex (*mm*^3^ = 113, *p* = 0.04), right caudate (*mm*^3^ = 1224, *p* = 0.0008) and bilateral cerebellum (left: *mm*^3^ = 584, *p* = 0.04; right 1: *mm*^3^ = 1104, *p* = 0.002; right 2: *mm*^3^ = 792, *p* = 0.01) (Figure 9 and Table 5).

**Table 5:** Regions that exhibited a relationship between heading disparity and the hemodynamic response.

Maxima coordinates (MNI)			Region name	Size (mm^3^)	Cluster-wise *p*-value
			
x	y	z			
-21.5	-5.9	48.0	Left superior frontal	708	0.0003
-8.2	8.9	53.1	Left superior frontal	230	0.0009
22.0	-1.4	56.9	Right superior frontal	778	0.0003
9.7	16.1	43.1	Right superior frontal	306	0.0003
-32.7	-41.6	41.1	Left superior parietal	1786	0.0003
41.7	-36.2	38.6	Right supramarginal	1594	0.0006
-26.1	-96.3	-1.3	Left lateral occipital	380	0.0003
-18.8	-86.7	-8.3	Left lateral occipital	285	0.0003
14.5	-91.9	15.3	Right lateral occipital	279	0.0003
-11.4	-90.8	-2.5	Left pericalcarine	305	0.0003
10.9	-86.0	-0.9	Right pericalcarine	704	0.0003
-28.7	17.5	9.3	Left insula	624	0.02
-40.1	-55.7	-11.5	Left fusiform	200	0.001
46.0	4.7	28.2	Right precentral	282	0.003
17.7	-75.9	-9.7	Right lingual	251	0.003
29.3	26.4	2.2	Right lateral orbitofrontal	113	0.04
16.0	19.0	1.0	Right caudate	1224	0.0008
-30.0	-47.0	-29.0	Left cerebellum	584	0.04
22.0	-65.0	-19.0	Right cerebellum	1104	0.002
42.0	-61.0	-31.0	Right cerebellum	792	0.01


## Discussion

Directional sense, or the ability to keep track of one’s orientation with respect to an environmental reference frame, is critical to remaining oriented in known environments and is part of the multi-faceted concept of sense-of-direction. The primary goal of this work was to elucidate the neural correlates of variation in environmental-scale spatial ability, as measured by the Relative Heading task and self-reported sense-of-direction. Specifically, we examined (1) the factors that contribute to variation in performance of the relative heading task, including self-reported sense-of-direction, (2) variation in brain structure related to variation in directional sense, and (3) the neural basis of directional sense. Below, we discuss the behavioral, structural and functional results, in relation to prior research.

### Behavioral Results

As in previous research on directional sense (Sholl et al., 2006; Burte and Hegarty, 2012, 2013, 2014), we found large individual differences in performance. Four predictors accounted for most of variation in direction sense: gender, self-reported sense-of-direction, familiarity, and directionality.

#### Gender

Males were more accurate, took less time to correctly respond, left fewer trials unanswered, and reported a better sense-of-direction than females. While gender differences are found in some but not all spatial tasks (Voyer et al., 1995; Montello et al., 1999; Coluccia and Louse, 2004) these results are consistent with results from other spatial tasks (e.g., Voyer et al., 1995; Coluccia and Louse, 2004), that likewise show gender differences in spatial tasks that involve knowledge acquired from direct experience in the environment. Critically, males and females did not differ in objective measures of familiarity, so differences in task accuracy were not likely due to familiarity differences. In both linear regression and linear mixed models, accuracy was significantly predicted by participant gender. These results are consistent with previous studies of the Heading Recall and Relative Heading task and indicate that male performance in some navigation tasks may in part be due to males’ greater facility in imagining, identifying and comparing allocentric directions.

#### Sense-of-Direction

Self-reported sense-of-direction was significantly correlated with task performance to a significant degree as reported previously (Burte and Hegarty, 2012, 2013, 2014), and predicted correct decision latencies. These results support the notion that the Relative Heading task assesses a skill, which we called “directional sense,” that underlies what individuals self-report as their sense-of-direction (Sholl et al., 2006). Following the conceptualization of sense-of-direction used to create the SBSOD scale (Hegarty et al., 2002), we propose that self-reported measures of sense-of-direction assess a set of skills that encompass how people orient themselves within known environments and with measures of spatial knowledge acquired from direct experience in the environment.

#### Familiarity

Even though participants were selected for high self-reported familiarity with the environment, accuracy was significantly predicted by mean near building accuracy. So even at the participant-level, one of our objective measures of familiarity was predictive of performance on the relative heading task. Previous studies (Sholl et al., 2006; Burte and Hegarty, 2012, 2013, 2014) had used self-reported ratings of familiarity to select participants and photographed locations for inclusion in their studies. While these ratings were correlated with performance in previous studies, in the current study, we found evidence that ability to identify the nearest building is more predictive than self-reported familiarity or building naming, on a participant-level. Our study points to the importance of measuring familiarity objectively, rather than relying on self-reported ratings.

At the level of individual trials, familiarity rating predicted both accuracy and correct decision latency while nearest building accuracy predicted accuracy. For the Relative Heading task, knowing where a building is located relative to other nearby buildings is more predictive than knowing its name, likely because understanding the layout of neighboring buildings is more closely related to the spatial reasoning required by the task than is knowledge of the building name, which relies on other (non-spatial) memory processes.

From these results, it seems likely that self-reported and objective measures both have their place in studies of environmental-scale spatial abilities. Self-reported familiarity might encompass more aspects of familiarity than objective measures, but objective measures might better assess a particular aspect of familiarity.

#### Directionality

Using linear mixed models, we found that accuracy was higher for specific target headings while correct decision latency was more accurate for specific orienting headings. In both cases, performance for north and west heading tended to be better (or faster) than for east and south headings, replicating previous research with this environment (Burte and Hegarty, 2012, 2013, 2014). This pattern is likely specific to the experiment location: the mountains are to north, and the neighborhood in which most participants live is to the west. In both cases, there are clear walkways on campus that open up vistas in these directions from the center of the campus. In contrast, the landmarks signifying the South and East directions (a lagoon and a beach) are occluded by other buildings from the center of campus, so there are no clear paths or vistas leading to these locations. This pattern is consistent with findings that pointing is more accurate from perspectives aligned with salient reference systems (e.g., Shelton and McNamara, 1997, 2001). In addition, the most familiar buildings to participants are clustered toward the north and west of campus (Figure 3C).

A novel finding of this study is that performance was influenced by participants’ physical location and orientation in the environment, even though they were instructed to imagine a different orientation, and they were lying in an MRI scanner. Specifically, we found an alignment effect such that performance was best when photographed locations were toward the participant’s feet while lying in the scanner and performance degraded when photographed locations were toward the head. These alignment effects are similar to those in the Heading Recall task (Sholl et al., 2006; Burte and Hegarty, 2012, 2013) in which performance was best when photographed locations were in front of participants, and worst when performance degraded when photographed locations were behind participants. Critically, previous research has shown that these alignment effects only occur when the participant is aware of the relationship between their body and the environment (Burte and Hegarty, 2014). These alignment effects are consistent with sensorimotor alignment effects, in which pointing is more accurate from an orientation that matches the individual’s physical orientation and degrades around the body (e.g., Kelly et al., 2007), or self-localization reaction times being related to angular discrepancy (Iachini and Logie, 2003).

Finally, heading disparity predicted correct decision latency, in that trials in which the orienting (imagined) and target headings were aligned were responded to the fastest. As in previous research (Burte and Hegarty, 2014), this effect was relatively weak compared to the sensorimotor alignment effect, supporting the conclusion that the original alignment effects found in the heading recall task were sensorimotor in nature. This partial alignment effect indicates that imagined headings are faster to compare when aligned, possibly because non-aligned headings need to be mentally rotated into congruence to be compared (cf. Shepard and Metzler, 1971).

#### Behavioral Summary

Despite the novelty and specialized nature of the Relative Heading task, performance on this task shares similarities with measures of a range of spatial skills. It shows gender differences in favor of males; sense-of-direction is predictive of performance on this task as well as other large-scale spatial tasks; and environmental familiarity impacts performance on this task. Finally, this task is subject to sensorimotor alignment effects, even when the orientation to be imagined is not one’s physical orientation. So, comparing allocentric headings is impacted by individual differences and environmental features similar to many other spatial skills.

### Structural Results

#### Sense-of-Direction

Hippocampal volume was related to self-reported sense-of-direction. Hippocampal volume has been related to path integration – a capacity that supports navigation (Chrastil et al., 2017), spatial strategy use (Bohbot et al., 2007; Konishi and Bohbot, 2013), cognitive mapping (O’Keefe and Nadel, 1978), and the ability to flexibly use spatial information acquired through route learning (Brown et al., 2014), so it follows that individuals with a better sense-of-direction might also have larger hippocampi. These results should be interpreted with caution as the subcortical analyses were not corrected for multiple comparisons, and SBSOD scores were not related to any of the hippocampal subfields. Instead, SBSOD scores were related to right presubiculum volume, a region that is involved in coding facing direction relative to the cardinal directions (Vass and Epstein, 2013). Since the knowledge and use of cardinal directions is part of what people conceptualize as a “good sense-of-direction,” perhaps the use of cardinal directions is associated with greater volume in the right presubiculum.

#### Relative Heading Performance

Performance on the Relative Heading task was associated with the left lateral orbitofrontal cortex, left precuneus, and right superior parietal. While not originally predicted, the lateral orbitofrontal cortex has been implicated in the suppression of previously rewarded responses (Elliott et al., 2000), which is a part of the Relative Heading and Heading Recall tasks. When first teaching participants about these tasks, their first instinct is to point toward the photographed location but they learn to inhibit that response and instead compare the two headings. It might be that individuals with larger lateral orbitofrontal cortices are better able to inhibit this pointing response, which contributes to greater accuracy on the Relative Heading task. In addition, left precuneus has been associated with computation of direction toward a goal, using egocentric coordinates (Chadwick et al., 2015). Whereas, right superior parietal activity has been associated with gender differences in representing space egocentrically (Grön et al., 2000). Perhaps individuals with a larger precuneus and superior parietal lobules are more experienced at relating the location and orientation of their body to the environment, which contributed to increased task performance. However, this interpretation is tentative, as the links between structure and function are rarely straightforward to interpret.

### Functional Results

We hypothesized that directional sense would be related to functional activation in areas that process task-relevant information. The Relative Heading task is composed of four processes: (1) imagining the orienting heading, (2) visually identifying the pictured location, (3) identifying the target heading, and (4) comparing the allocentric headings. However, note that not each of these processes should necessarily be expected to differ as a function of heading disparity.

#### Identifying Allocentric Headings

Both the orienting and target headings (processes 1 and 3) must be identified before they can be compared. The hippocampus and RSC/PC, with their surrounding areas, have been implicated in allocentric coding and likely interact (Ekstrom et al., 2014). Since the RSC/PC is more involved in orientation changes without self-motion (Gomez et al., 2014), we hypothesized that the RSC/PC might show activation related to heading disparity. However, these areas did not show a linear response with the difference between the headings. This may be because, while these areas were involved in allocentric coding, more processing was not needed as heading disparity increased. Each trial involved the same load in terms of allocentric coding as two headings needed to be identified for each trial.

#### Visual Processing of Pictured Locations

While the orienting heading is presented via text, the target heading must be identified from a photograph (process 2). Four areas associated with visual processing showed increased functional activation with heading disparity: lateral occipital cortex, which is involved in object perception (Grill-Spector et al., 2001), the pericalcarine cortex, which is the primary visual cortex, along with the lingual gyrus and fusiform, which are involved in visual processing and reading (Mechelli et al., 2000). It is possible that these visual processing areas showed a linear response with the difference between the headings, because the visual processing and imagery needed to compare the headings scale linearly. For example, if 0° deviations between headings are easier to determine (the behavioral data suggests that this is true because decision latencies were shorter for 0° deviations), then participants might focus less attention to the photographed target headings and orienting heading text, and/or visualize less when comparing the headings. However, if 180° deviations between headings are more difficult to determine, then participants might attend more to the photographs and text, visualize the environment or nearby buildings, and/or imagine turning or moving in the environment. So, in this task, visual processing increases with the angular deviation between the headings.

#### Comparing Allocentric Headings

Once the photographed location has been identified, participants need to compare the orienting and target heading. This can be done using an egocentric (i.e., imagining turning the body within the environment) and/or allocentric (i.e., east is 90° right from north) perspectives (Burte and Hegarty, 2013). Given that the RSC/PC has been implicated in translating between egocentric and allocentric coding (Byrne et al., 2007; Epstein, 2008), we predicted that the RSC/PC would be involved in not only the coding of allocentric headings (steps 1 and 3) but also in heading comparison (step 4). We observed several clusters that might be related to the comparison process: superior frontal gyrus, superior parietal lobule, supramarginal gyrus, and lateral orbitofrontal cortex. The posterior parietal and frontal structures are involved in body-centered spatial coding (for a review see Galati et al., 2010), indicating that participants might have related heading disparity to the response buttons in a body-centered manner. Similar to the structural findings, the lateral orbitofrontal cortex is involved in suppression of previously rewarded responses (Elliott et al., 2000) and this suppression effect increased with heading disparity. Contrary to our prediction, we did not observe activation in RSC/PC, possibly because parieto-frontal regions carry out the comparison by translating the allocentric coding done in the RSC/PC into ego-relative coordinates (Filimon, 2015).

#### Unpredicted Results

Three areas showed functional activation related to heading disparity that fell outside our focus on hippocampal and RSC/PC-connected areas: caudate, precentral gyrus, and cerebellum. The caudate tends to be active in spatial tasks that required delayed motor responses (Postle and D’Esposito, 1999). In the Relative Heading task, the button-press response must be delayed until after the picture heading is presented and the comparison of headings has occurred. Given that decision latencies increase with increasing heading disparity, it follows that activation in the caudate should also increase with heading disparity. The significant cluster in the right precentral gyrus was likely associated with the button-press response, although activity in this area might have been associated with imagined motions, as increasing heading disparities would require increased imagined turning. Furthermore, significant clusters were found in the lobes of the cerebellum, spanning Crus I and lobules IV, V, and VI. Right Crus I has been implicated in sequence-based navigation (i.e., navigation based on egocentric representations; Iglói et al., 2014), Crus I and lobules VI were associated with working memory, and lobule V with finger tapping (Stoodley et al., 2012). These nuclei have been associated with highly relevant processes, in particular, accounting for heading disparity in the context of understanding self-motion (Baumann et al., 2015) and tracking rotational self-motion (Chrastil et al., 2017). The present task may rely on the same computational machinery, but in service of computing stationary heading disparity, rather than parsing self-motion.

## Conclusion

We have demonstrated that a shared network, featuring many regions that have previously been associated with spatial reasoning including superior frontal gyrus, superior parietal lobule, supramarginal gyrus, lateral orbitofrontal cortex, and caudate, is active in comparing headings. While the retrosplenial cortex and hippocampus have been frequently implicated in the coding of allocentric headings, this work revealed frontal and parietal regions were involved in comparing headings that the RSC/PC and hippocampus coded. Moreover, in line with previous work, we found large individual and gender differences in task performance, as well as in self-reported sense-of-direction. These individual differences may also relate to structural differences in relevant areas including superior parietal cortex. Thus, this work has helped to further our understanding of variation in directional sense.

## Data Availability

The raw data supporting the conclusions of this manuscript will be made available by the authors, without undue reservation, to any qualified researcher.

## Author Contributions

All authors listed contributed to designing this study. HB and BT developed the experimental stimuli and materials, oversaw data collection, and completed data analysis. HB drafted the initial version of this manuscript as part of her Ph.D. dissertation. HB and BT wrote, and MH edited this manuscript.

## Conflict of Interest Statement

The authors declare that the research was conducted in the absence of any commercial or financial relationships that could be construed as a potential conflict of interest.

## Footnotes

1 In pilot studies, we attempted to use photographs for to specify the orienting heading as well as the target heading, but participants tended to misinterpret the task as a task of pointing from the location presented in the orienting heading towards the location in the target heading. (Experiment 1 in Burte and Hegarty, 2014). In order to force participants to compare the two headings, we moved to using a text description of the orienting heading, which specified a direction but not a location.

2 http://psychtoolbox.org

3 http://freesurfer.net/

4 http://www.fil.ion.ucl.ac.uk/spm/

5 Due to an error in the prescreening, 25 participants did not respond to building naming and nearest building tasks for one east-facing photographed location. These trials were excluded from analysis (1.4% of trials).
